# Evaluation of artificial intelligence based prosthetic innovation on dental implants: A database research

**DOI:** 10.6026/973206300221401

**Published:** 2026-03-31

**Authors:** Jay Gohil, Subachander Prabhakaran, Nishtha Agrawal, Mithun Ganesh, Sree Ram Subba Reddy Gudimetla, Raghavendra Nagappa, Rahul Tiwari

**Affiliations:** 1 Department of Prosthodontics, Crown & Bridge, K.M. Shah Dental College & Hospital, Sumandeep Vidyapeeth (Deemed to be University), Waghodia, Vadodara, Gujarat, India; 2 Department of Prosthodontics and Crown and Bridge, Meenakshi Ammal Dental College and Hospital, Chennai, Tamil Nadu, India; 3 Department of Prosthodontics, Government College of Dentistry, Indore, Madhya Pradesh, India; 4 Department of Oral and Maxillofacial Surgery, Sri Sai College of Dental Surgery, Vikarabad, Telangana, India; 5 Department of Oral Maxillofacial surgery, Sree Mithra Dental and MaxFace Specialists, Tanuku, Andhra Pradesh, India; 6 Department of Periodontics and Oral Implantology, College of Medical Sciences, Bharatpur, Chitwan, Nepal; 7 Department of Dental Research Cell, Dr. D. Y. Patil Dental College & Hospital, Dr. D. Y. Patil Vidyapeeth (Deemed to be University), Pimpri, Pune 411018, Maharashtra, India

**Keywords:** Artificial intelligence, deep learning, dental implant, panoramic radiograph, prosthodontics

## Abstract

Accurate radiographic identification of dental implant systems remains challenging in clinical practice when implant documentation is unavailable and manual interpretation of panoramic radiographs is time-consuming and prone to error. Participants and methods deep learning performance was retrospectively evaluated in a database study for automated dental implant system recognition on panoramic radiographs using a labelled institutional image set. Model evaluation was based on accuracy, precision, recall and F1-score on a held-out test set. The top model reached a high diagnostic precision and only low rate of confounding between visually similar implant systems. Thus, we show the potential of using AI-supported implant recognition in prosthetic rehabilitations, especially when no implant documentation is available.

## Background:

Radiographic recognition of dental implant systems is clinically indispensable for prosthetic maintenance, selection of components, peri-implant diagnosis and planning of revision. In "real life," lack of data on the implant brand is common, as well it being overlooked in records or transferred between colleagues/clinics, rendering radiographic identification a useful skill. Nevertheless, manual assessment from panoramic images is laborious and potentially inaccurate when implant silhouettes are alike. Deep learning, including convolutional neural network (CNN), has achieved excellent performance in dental image classification and detection such as implant type, number and radiographic features recognition [1]. More recent studies have achieved high accuracy for automated implant system classification using large multicenter radiographic datasets and as well as validated CNN pipelines [2, 3]. Moreover, deep learning approaches have shown consistent detection of implants and implant- related findings in dental radiology domain [4, 5]. Therefore, it is of interest to report and evaluate the performance of deep learning-based models for automated dental implant system recognition using retrospective panoramic radiographic data.

## Materials and Methods:

Methods this was a retrospective database study that obtained panoramic radiographs from an institutional digital radiology archive spanning the period of January 2019 through December 2025. Images were included if a dental implant fixture was visible in at least one image and the implant system could be traced from operatory notes. Images with moderate to severe motion interference, inadequate exposure or implant obscured areas were discarded. Four popular implant systems (referred to as Brand A-D) were selected in a balanced manner and according to their availability. Radiographs were anonymized, saved as standardized files and pre-processed. Implant regions were localized using manual bounding boxes by 2 two trained clinicians; disagreements were determined by consensus. The dataset was divided at the patient level into training (70%), validation (15%) and test (15%) data sets to prevent leakage. We further developed a CNN-based classification pipeline for transfer learning, based on pretrained architectures. Augmentation (rotation, contrast shift and scaling) was applied to the data to prevent overfitting. The main outcome was classification performance on the test set. Secondary measures were accuracy, recall, F1-score and patterns of confusion matrix. The model was evaluated using standard classification metrics of supervised learning and results were presented descriptively as a proof-of-principle database validation study.

## Results:

Deep learning models achieved the high performance results for implant system identification based on panoramic radiographs. The best results overall were obtained by the optimized VGG16-based classifier, with a 98.9% accuracy and balanced precision (97.8%) and recall (96.9%). DenseNet-121 and EfficientNet-B0 also performed well with the accuracy rate that exceeded 96.300%. ResNet-50 had slightly lower scores but was still clinically acceptable. In general terms CNN systems efficiently discriminated implant systems according to radiographic morphology Table 1 (see PDF). The correct classified values of all four implant systems recorded in the confusion matrix were generally high. Misclassification tended to involve Brand B versus Brand C and Brand D versus Brand C due to shared radiographic fixture silhouette features. Brand A exhibited the best stability, as it was only one sample misclassification. The low percentage of cross-brand errors reflects the high degree of discriminative power and supports clinical feasibility for automated implant recognition in retrospective radiograph databases Table 2 (see PDF).

## Discussion:

In the current retrospective database study, it was shown that deep learning can correctly categorize dental implant systems by panoramic radiographs with high diagnostic accuracy. The highest performing model reached an accuracy of 98.9% and had high precision and recall, suggesting potential value for automated implant system detection in clinical workflows of prosthetic dentistry. The feasibility of recording identification by means of deep learning has been supported by recent evidence, involving also sizeable datasets and multicenter validation strategies. One multicenter study concluded that the identification of implant systems can be performed by DL models from various radiographic inputs, suggesting generalizability when training was quite heterogeneous [6]. Noteworthily, strong CNN-based implant brand classification models were also reported by other studies with high accuracy and the present study's result suggests that traditional deep learning architectures are still effective when structured pre-processing (balancing the dataset) is performed as well as balancing label categories accordingly [7]. In addition to being brand-specific highlighter detection from panoramic radiographs, deep learning has been used for implant detection and numbering on the same type of radiograph these works show that AI can be used in an implant-centric manner for more than just classification but workflow as a whole [8]. Regarding prosthodontic point of view, the automatic implant-recognition can directly minimize the clinical uncertainty in choosing abutments and performing also peri-implant complications treatments and any reconstructive-prosthetic approach. This is particularly pertinent in the absence of available implant documentation, which is often seen in referral cases. AI systems could also help in clinical decision-making by facilitating fast categorization of implanted device prior to any further imaging or invasive examination. A side from implant recognition, AI has been employed in recent research for radiographic tasks associated with peri-implantitis, such as segmenting and detecting the peri- implant bone loss. This suggests that implant-related AI could be developed as an all-in-one system with a feature for screening of the implants and pathology (e.g., repositioning) [9]. More general evidence on the use of deep learning for implants as well as various radiographic findings originating from dental radiology has been published to support its value for general diagnostic support [10]. However, the substantial limitations still remain despite these positive outcomes. First, this was a single-center retrospective dataset and we might have external generalizability. Secondly panoramic radiographs are inherently deformed and superimposed [7] and behavior could be different in periapical radiographs or CBCT [11, 12-13]. Thirdly the investigation only evaluated four implant systems; additional brands would likely reduce clinical utility and accuracy unless based on significantly larger datasets. Finally, AIs model should be interpretable and validated, to avoid hidden bias when used in clinical practice safely. Prospective, multicenter external validation and prospective testing in real-world practice are needed in future studies as well as the combination of object detection models with classifiers to achieve a fully automated localisation plus recognition of implants. Integrating machine learning with AI-based implant identification using established prosthetic platforms could significantly contribute to the further development of digital prosthodontics [14, 15].

## Conclusion: 

Deep learning models may classify dental implant systems on panoramic radiographs with high precision in retrospective database scope. Implant recognition software may improve prosthetic workflows by decreasing manual diagnostic burdens in the event that implant records are not available. External validation at multiple centers is necessary prior to routine clinical implementation.

## Advancement to knowledge:

This database study provides contemporary evidence (2020-2025) that transfer learning-based convolutional neural networks can achieve very high diagnostic accuracy for automated dental implant system recognition on panoramic radiographs, with minimal cross-brand misclassification, thereby supporting the feasibility of AI-assisted implant identification in prosthodontic workflows when clinical documentation is unavailable.
